# 1, 25-dihydroxyvitamin D_3,_ a potential role player in the development of thyroid disorders in schizophrenics

**DOI:** 10.12669/pjms.326.11157

**Published:** 2016

**Authors:** Arif Malik, Shamaila Saleem, Muhammad Abdul Basit Ashraf, Mahmood Husain Qazi

**Affiliations:** 1 Dr. Arif Malik, PhD, Institute of Molecular Biology and Biotechnology (IMBB), The University of Lahore, Lahore, Pakistan; 2 Dr. Shamaila Saleem, MBBS, M.Phil, Institute of Molecular Biology and Biotechnology (IMBB), The University of Lahore, Lahore, Pakistan; 3 Dr. Muhammad Abdul Basit Ashraf, MBBS, Institute of Molecular Biology and Biotechnology (IMBB), The University of Lahore, Lahore, Pakistan; 4 Dr. Mahmood Husain Qazi, PhD. Centre for Research in Molecular Medicine (CRiMM), The University of Lahore, Lahore, Pakistan

**Keywords:** Schizophrenics, Autoimmune thyroid diseases, GSH, NOS, Homocysteine

## Abstract

**Objective:**

The present study was designed to assess the role of vitamin-D, in the development of autoimmune thyroid dysfunction in newly diagnosed schizophrenics.

**Methods:**

For the present study 100 patients and 100 controls were screened out and studied for their thyroid antibodies, GSH, homocysteine, NOS and vitamin D levels by appropriate protocols to assess the underlying mechanism involved in the schizophrenics susceptible to autoimmune thyroid diseases.

**Results:**

The results of the present study depicted that in schizophrenics, levels of cytokines like IL-6 (7.98±0.67 pg/ml), TNF-α, (40.76±6.98 pg/ml), homocysteine (16.98±1.09 µmol/L), Tg-Ab (30.93±3.87 IU/L), TPO-Ab (10.33±1.78 IU/L) and TSHr-Ab (3.76±0.055 IU/L) increased whereas, those of Vit-D (12.76±0.99 pmol/L), NOS (5.99±0.87 IU/L), GSH (4.48±.965 µg/dl) and NO (16.87±3.98 ng/ml) were decreased in the patients as compared to healthy control subjects.

**Conclusion:**

Vitamin-D in schizophrenia is involved in augmentation of hyperhomocysteinemia, inflammation, oxidative stress and thyroid antibodies, thereby playing a significant role not only in induction of schizophrenic symptoms but may also result in autoimmune thyroid diseases. Thus, earlier detection and rectification of its levels are helpful to limit the miseries of schizophrenia.

## INTRODUCTION

In the recent years, it has been shown that vitamin D (Vit-D) is not only important in mineral homeostasis but also in the prevention of cancer, cardiovascular diseases and of particular interest, autoimmune thyroid diseases and schizophrenia. Vitamin D is obtained endogenously from sunlight and exogenously from milk products, salmon, tuna and sardines.^1^ The other factors which influence vitamin D concentrations are old age, genetic make-up, female gender and higher skin pigmentation.^2^,^3^ Furthermore, the individuals having neurocognitive decline spend less time outside and have poor nutrition, both of which result in limiting Vit-D levels.^1^ Its final active form [1, 25(OH)_2_ D_3_] is achieved after two sequential enzymatic hydroxylations, firstly in liver and then in kidney mediated by CYP27B1.^4^ Previously, vitamin D concentrations in brain were considered to be dependent on its plasma levels until the ideas of expression of CYP27B1 and vitamin D receptors (VDR) in different tissues were introduced. The brain has an ability to form an activated form of vitamin-D [1, 25(OH)_2_D_3_] in hypothalamus and substantia nigra, while VDR and catalytic enzymes are expressed in human cortex and hippocampus, which are important in complex planning, thought processing and new memories synthesis.^5^,^6^

Vitamin-D plays an important role in neuroprotection by regulating the levels of neurotropin-3, nerve growth factor (NGF), glial cell derived neurotrophic factor (GDNF) and of particular concern thyroid antibodies [thyroid peroxides antibodies (TPOAb), thyroglobulin antibodies (TgAb) and thyroid stimulating hormone receptor antibodies (TSHrAb)], nitric oxide synthase (NOS), homocysteine (Hcy) and glutathione (GSH).^7^-^10^ Vitamin D has immunomodulator, anti-inflammatory and anti-oxidative properties. Reduction in vitamin D levels may result in an increase in auto-antibodies, helper T-cells activity and homocysteine levels, while a decrease in glutathione and nitric oxide synthase levels. These effects provide an interesting link between deficient vitamin-D levels, autoimmune thyroid diseases and neurocognitive problems.

## METHODS

One hundred patients of Schizophrenia diagnosed were screened out from the Social Security Hospital Lahore and 100 healthy individuals were selected as controls. All the experimental work was performed in the Institute of Molecular Biology and Biotechnology, University of Lahore, Lahore.

### Inclusion criteria

Newly diagnosed schizophrenia patients were selected which were not on any type of medication. The patients selection criteria of the present project was based on “Positive and Negative Syndrome Scale” (PANSS) and the “Clinical Global Impression-Severety scale” (CGI-S) being used to evaluate disease activity.

### Exclusion criteria

The subjects with the history of taking drugs (Including alcohol and cigarette), pre-diagnosis medications (e.g. antiparkinsonian/antipsychotic), were excluded. None of the controls were on any medication, history of chronic infections, malnutrition syndrome and metabolic dysfunction (Such as diabetes mellitus, liver diseases, cancer etc.) that could interfere with their oxidative metabolites and thyroid hormone status.

### Biochemical analysis

TPO, Tg and TSHrAb were appraised with the help of human ELIZA kit (BioVendor). NOS was estimated by the ELISA kit manufactured by Cayman Chemicals. IL-6 evaluation was done with quantitative ELISA after samples were drawn and stored at appropriate temperature (R&D Systems, Minneapolis, MN, USA).^11^ NO was estimated by using Griess reagent method as explained by the Bories and Bories.^12^ Tumor necrosis factor-alpha (TNF-α) was determined by ELISA kits (Affimatrix, Japan) and was then expressed in units (pg/ml). Homocysteine was estimated by using amino acid analyzer. Vitamin-D was determined by the ELISA kit method of ALPCO, USA. GSH was determined by the protocol mentioned by Moron *et al*.^13^

## RESULTS

The levels of interlukin-6 (IL-6) and tumor necrosis factor-alpha (TNF-α) differed significantly (p=0.0195) from the control (Table-I). The higher levels of IL-6 and TNF-α (7.98±.67 and 40.76±6.98 pg/ml) were recorded in the newly diagnosed schizophrenia patients. The levels of both these variables were also significantly higher than the control regarding sex. Higher levels of IL-6 and TNF-α were noted in males than those in females (8.26±0.56 in males vs 7.52±0.34 in females; 42.25±6.35 in males vs 39.27±2.23 in females respectively). The mean values of interlukin-6 (IL-6) and tumor necrosis factor-alpha (TNF-α) in control were 4.87±0.45 and 21.76±3.87 respectively. The levels of Tg-Ab, TPO-Ab and TSHrAb antibodies increased significantly (30.93±3.87, 10.33±1.78 and 3.76±0.055 pg/ml) in the newly diagnosed patients of schizophrenia. The levels of these variables were significantly higher in both sexes while highest level of Tg-Ab was recorded in males (31.22±4.25 pg/ml) than those in females (30.65±3.65 pg/ml). Whereas, the levels of TPO-Ab and TSHr-Ab were recorded higher in females (13.40±1.01, 5.63±0.3 pg/ml) when were compared with those of males (7.26±0.98, 1.89±0.09 pg/ml). The mean values of Tg-Ab, TPO-Ab and TSHr-Ab in controls remained (21.87±1.87, 6.81±1.03 and 1.44±.017 pg/ml) respectively.

**Table-I:** Biochemical profile of schizophrenics versus controls.

Variables	Schizophrenics vs Control (MEAN±SD)				P-values (<0.05)

	Control	Mean value	Male	Female	
IL-6 (pg/ml)	4.87±0.45	7.98±0.67	8.26±0.56	7.52±0.34	0.0195
TNF-α (pg/ml)	21.76±3.87	40.76±6.98	42.25±6.35	39.27±2.23	0.0114
Tg-Ab (IU/L)	21.87±1.87	30.93±3.87	31.22±4.25	30.65±3.65	0.009
TPO-Ab (IU/L)	6.81±1.03	10.33±1.78	7.26±0.98	13.40±1.01	0.0034
TSHr-Ab (IU/L)	1.44±0.017	3.76±0.055	1.89±0.09	5.63±0.03	0.019
Vit-D (pmol/L)	17.87±1.48	12.76±0.99	14.27±1.23	11.25±0.91	0.0032
Homocysteine (μmol/L)	5.87±1.987	16.98±1.09	14.26±0.56	19.70±0.85	0.016
NOS (IU/L)	7.87±1.87	5.99±0.87	6.54±0.691	5.44±0.994	0.0176
GSH (μg/dl)	9.06±1.75	4.48±0.965	4.13±1.089	4.76±1.78	0.0376
NO (ng/ml)	23.27±3.87	16.87±3.98	17.86±1.87	15.88±1.99	0.0064

Levels of Vit-D differed significantly different (p=0.0032) when compared with controls (Table-I). The levels of Vit-D decreased (12.76±0.99 pmol/L) in the patients with the newly diagnosed schizophrenia. The levels of Vit-D significantly decreased in both sexes despite the fact that highest decrease in Vit-D was in females (11.25±0.91 pmol/L). The mean value of Vit-D in controls was (17.87±1.48 pmol/L). The results from Table-1 shows that the levels of GSH and NO differed significantly (p=0.0376) when compared with controls. The levels of GSH and NO decreased significantly (4.48±.965 µg/dl, 16.87±3.98 ng/ml) in the patients with schizophrenia. These values were significant for both sexes. The levels of GSH and NO were recorded 4.13±1.089µg/dl, 17.86±1.87 ng/ml in males respectively and 4.76±1.78 µg/dl, 15.88±1.99 ng/ml respectively in females. The mean values of these variables in controls remained 9.06±1.75 µg/dl, 23.27±3.87ng/ml respectively. The levels of NOS differed significantly (p=0.0176) when compared with the controls. The concentration of NOS decreased significantly (5.99±0.87 IU/L) in the patients of newly diagnosed schizophrenia. Higher decrease in NOS can be seen in females (5.44±0.994 IU/L) as compared to that in males (6.54±0.691 IU/L). The mean value of NOS in controls was recorded as 7.87±1.87 IU/L. The results presented in Table-1 states that the levels of homocysteine were significantly different (p=0.016) from the controls. The levels of homocysteine increased markedly (16.98±1.09 μmol/L) in the patients of newly diagnosed schizophrenia. However, the trend remained similar in both sexes whereas, highest increase in the homocysteine could be seen in the females (19.70±0.85 μmol/L) with respect to the males (14.26±0.56 μmol/L). The mean value of homocysteine in controls was recorded as 5.87±1.987 μmol/L.

## DISSCUSSION

The present study showed that vitamin D levels were deficient in schizophrenics and had an inverse correlation between vitamin D levels, thyroid antibodies, inflammatory markers and homocysteine levels while positive correlation between nitric oxide, nitric oxide synthase and glutathione. The reduced vitamin D concentrations are believed to cause dendritic cell mediated activation of helper T cells-2 (Th-2) which release inflammatory markers i.e. interleukin-6 and tumor necrosis factor-alpha, resulting in increased adaptive immunity and specifically in thyroid gland. The levels of thyroid antibodies (TPOAb, TgAb and TSHrAb) are increased leading to autoimmune thyroid diseases (AITDs) including Graves’ disease and Hashimoto’s thyroiditis,^14^,^15^ as both types of AITDs were observed in the present study having inverse correlation between vitamin-D and thyroid antibodies (Vit-D Vs TPOAb, r=-0.693, Vit-D Vs Tg, r=-0.345 and Vit-D Vs TSHrAb, r=-0.455).

**Fig.1:**
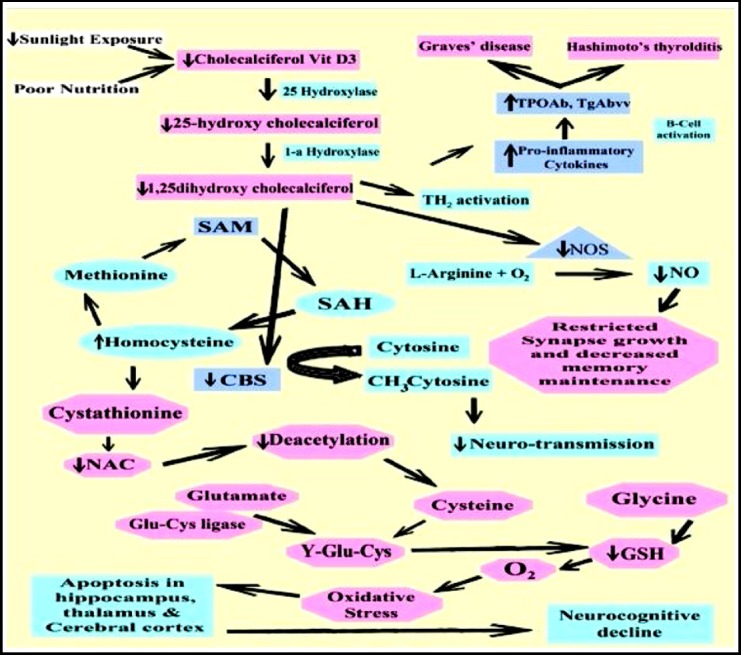
In schizophrenics, due to reduce sunlight exposure and poor nutrition there is deficiency of active form of vitamin D (1, 25 dihydroxycholecalciferol) resulting in increase in inflammation by activation of Th2 causing raised immune response leading to increase in thyroid antibodies titer. This increased titer results in autoimmune thyroid diseases i.e. Hashimoto`s thyroiditis and Graves` disease. On the other hand, reduced active vitamin D_3_ levels result in not only decrease in nitric oxide synthase (NOS) but also cystathionine β synthase (CBS) leading to reduction in nitric oxide (NO) and increase in homocysteine levels respectively. The reduced NO levels result in restricted synapse growth and decreased memory maintenance while hyperhomocysteinemia leads to cognitive decline as methylation of neurotransmitter is decreased due to decreased S adenosylmethionine (SAM). Decreased processing of homocysteine into glutathione (GSH) causes oxidative insult results in apoptosis in hippocampus, thalamus and cerebral cortex leading to neurocognitive decline.

Homocysteine, a sulfur containing amino-acid is either methylated to form methionine and then into S-adenosylmethionine (SAM) or trans-sulfurated into cystathionine in the presence of cystathionine β-synthase (CBS). Vitamin D levels regulate homocysteine concentrations by controlling the CBS activity and reduction in vitamin D levels induces inhibition of CBS activity leading to hyperhomocysteinemia having a negative impact on homocysteine and SAM balance,^4^,^16^ as shown in Fig.1. The present study also depicted an inverse correlation between vitamin D and homocysteine levels (Vit-D Vs Homocysteine, r= -0.523). Defective SAM synthesis may results in impaired myelin formation/restoration and neurotransmitter yield leading to interference with neurocognitive function.^17^ As trans-sulfuration of homocysteine is reduced due to deficient vitamin D, there is also reduction in glutathione levels because homocysteine is ultimately trans-sulfurated into glutathione^9^ and the current study also demonstrates a positive correlation between vitamin D and glutathione (Vit-D Vs Glutathione, r=0.413). The reduced glutathione levels result in decreased processing of pro-oxidants and decreased levels of antioxidants leading to oxidative disturbance, as illustrated in Fig.1. This causes increase in nuclear damage and apoptosis especially in cerebral cortex, thalamus, amygdala and hippocampus thereby resulting in ventricular enlargement leading to schizophrenic symptoms.^18^

It has been proposed that vitamin D is inversely correlated with nitric oxide synthase (NOS) levels, but the current study revealed a positive correlation between these two variables (Vit-D Vs NOS, r=-0.336). A similar relationship was observed by Rockett *et al*. (1998).^19^ Nitric oxide (NO) is formed from L-arginine and oxygen in the presence of NOS and the reduction in NOS levels due to deficient vitamin D levels result in decreased levels of NO which is important in memory maintenance, cognitive abilities and synaptic plasticity mainly involved the postsynaptic regulation of actin cytoskeleton through cGMP-PKG cascade,^20^,^21^ as demonstrated in Fig.1. Vitamin D regulates the levels of thyroid antibodies, oxidative stress and inflammatory markers thus, by controlling its levels within normal range the deleterious effects of deficient Vit-D can be mitigated.

### Limitation of the study

Although the research has achieved its objective but there were some unavoidable limitations. First, this research was conducted only on a small size of population. Due to financial constraints, the study was under-powered; therefore, to generalize the results for larger groups. Further studies should have involve more participants at different levels to confirm the findings of the study.

## CONCLUSION

Current study shows the significant role of vitamin D (Vit-D), nitric oxide (NO) and homocysteine (Hcy) in the development of autoimmune thyroid disorder in schizophrenics. Decreased levels of (Vit-D), and increased levels of (NO) and homocysteine (Hcy) may result in increased production of several cytokines causing raised thyroid antibodies titer leading to autoimmune thyroid dysfunction in schizophrenics. The results of the present study have revealed that individuals with vitamin D deficiency are more prone to develop thyroid disorders in patients suffering from schizophrenia than people with normal levels of vitamin D.
